# Thyroid Disrupting Effects of Old and New Generation PFAS

**DOI:** 10.3389/fendo.2020.612320

**Published:** 2021-01-19

**Authors:** Francesca Coperchini, Laura Croce, Gianluca Ricci, Flavia Magri, Mario Rotondi, Marcello Imbriani, Luca Chiovato

**Affiliations:** ^1^ Laboratory for Endocrine Disruptors, Unit of Internal Medicine and Endocrinology, Istituti Clinici Scientifici Maugeri IRCCS, Pavia, Italy; ^2^ Department of Internal Medicine and Therapeutics, University of Pavia, Pavia, Italy; ^3^ Department of Public Health, Experimental and Forensic Medicine, University of Pavia, Pavia, Italy

**Keywords:** perfluorinated alkylated substances, perfluorinated alkylated substances alternatives, endocrine disruptor, perfluorooctanoic acid and perfluorooctane sulfonic acid, thyroid, hypothyroidism, GenX, pregnancy

## Abstract

Per- and polyfluoroalkyl substances (PFAS) represent a group of synthetic compounds widely used in industry plants due to their low grade of degradation, surfactant properties, thermic and flame resistance. These characteristics are useful for the industrial production, however they are also potentially dangerous for human health and for the environment. PFAS are persistent pollutants accumulating in waters and soil and recoverable in foods due to their release by food packaging. Humans are daily exposed to PFAS because these compounds are ubiquitous and, when assimilated, they are difficult to be eliminated, persisting for years both in humans and animals. Due to their persistence and potential danger to health, some old generation PFAS have been replaced by newly synthesized PFAS with the aim to use alternative compounds presumably safer for humans and the environment. Yet, the environmental pollution with PFAS remains a matter of concern worldwide and led to large-scale epidemiological studies both on plants’ workers and on exposed people in the general population. In this context, strong concern emerged concerning the potential adverse effects of PFAS on the thyroid gland. Thyroid hormones play a critical role in the regulation of metabolism, and thyroid function is related to cardiovascular disease, fertility, and fetal neurodevelopment. *In vitro*, *ex vivo* data, and epidemiological studies suggested that PFASs may disrupt the thyroid hormone system in humans, with possible negative repercussions on the outcome of pregnancy and fetal-child development. However, data on the thyroid disrupting effect of PFAS remain controversial, as well as their impact on human health in different ages of life. Aim of the present paper is to review recent data on the effects of old and new generation PFAS on thyroid homeostasis. To this purpose we collected information from *in vitro* studies, animal models, and *in vivo* data on exposed workers, general population, and pregnant women.

## Introduction

Per- and polyfluoroalkyl substances (PFAS) are a family of synthetic chemicals widely used in industry. The first PFAS were invented in the 1930s and used in the production of non-stick and waterproof coatings, according to the Interstate Technology & Regulatory Council (ITRC). Nowadays, more than 3,000 synthetic compounds are classified as PFAS. They have wide-ranging applications, such as Teflon, food packaging, grease-resistant microwave-food bags, carpets that resist stains, stain-resistant furniture, fire foaming, and pipes and wires that resist corrosion (1, 2).

PFAS are organic substances composed by a chain of carbon atoms (in variable number, from 4 to 16) linked to fluorine atoms. A functional group at the end of the chain essentially distinguishes the various compounds of this family from one another. PFAS are generally classified in long- and short-chain compounds due to the presence of more than six (long-chain) or less than eight (short-chain) carbons (1, 3, 4). Carbon-fluorine bonds are among the strongest chemical bonds in organic chemistry. Thank to this bond, PFAS resist degradation and persist not only in the environment, but also in the human and animal body from which they are difficult to be eliminated. PFAS are not flammable and not corrosive, they do not burn, degrade, or react with other chemicals. In general, PFAS chemicals have a hydrophobic head and a hydrophobic tail (1, 3, 4).

PFAS have been frequently reported to contaminate surface water, groundwater, and soil. Indeed, given their persistence and mobility, they do not break down in environmental matrices and can move through soil to drinking water (2, 5, 6). This implies that if PFAS release will not be stopped, they will continue to accumulate in the environment, drinking water, and food, thus becoming global environmental pollutants potentially threating the human health and wildlife (2). As assessed by a biomonitoring approach, the concentration of PFAS in blood samples follows an overall order as: serum > plasma > whole blood (2). Geographical variations in blood PFAS concentration results from dietary intake, drinking water, dust, and industrialization level of the investigated area. In human blood, the concentration of PFAS appears to be associated with age, gender, and other factors, including dietary habits, lifestyle, family income, education level, social status, and body mass index (2). PFAS levels in human milk are overall much lower than those in human blood, likely due to low transfer rate of these chemicals from mother’s blood to breast milk. To date, studies remain limited on the use of human hair or nails as a non-invasive approach for PFAS biomonitoring (2).

PFAS can have several detrimental effects of endocrine function including the thyroid one. PFAS were recognized, among the potential adverse role on human health, as endocrine disruptors. In our previous review we reported the main findings regarding the potential adverse effects of PFAS on thyroid gland taking into account studies until 2017, evidencing controversial data about PFAS action on thyroid hormones both in *in vitro* studies and on different type of subjects (7). Thyroid hormones (TH) are involved in several biological processes, which include regulation of energy expenditure, growth, and neurodevelopment, starting from intrauterine life throughout infancy. Moreover, they regulate metabolic processes also in adult life (8–10).

During fetal life, thyroid hormones are crucial for normal brain development, being essential for orchestrating the processes of neurogenesis, migration, synaptogenesis, and myelination (8–10). Indeed, insufficient thyroid hormone levels at critical times of human neurodevelopment can induce long-term intellectual and behavioral impairments (8–10). Moreover, after birth and during all stages of infancy and adult life, TH regulate metabolism primarily through actions in the brain, white fat, brown fat, skeletal muscle, liver, and pancreas. TH are secreted through the hypothalamic–pituitary–thyroid axis (HPT axis). Briefly, the hypothalamus synthetizes and releases the thyrotropin-releasing hormone (TRH) into the pituitary portal circulation. TRH stimulates the release of thyrotropin (TSH) from the anterior pituitary, which in turn stimulates the synthesis and release of thyroid hormones, 3,5,3′,5′-tetraiodothyronine (T4) and 3,5,3′-triiodothyronine (T3) by the thyroid gland (8). T4 is exclusively secreted by the thyroid gland whereas nearly 80% of the circulating T3 results from peripheral conversion of T4 through the activity of the deiodinase enzymes located in target tissues, including the central nervous system (11). The action of the three different deiodinase enzymes deiodinase leads to either activation or inactivation of the thyroid hormones allowing a fine-tuning of thyroid hormone action at the tissue, cellular, and subcellular level by regulating the local availability of active thyroid hormone T3. The excess of thyroid hormone is commonly referred to as thyrotoxicosis/hyperthyroidism, and it promotes a hypermetabolic state characterized by increased resting energy expenditure, weight loss, increased lipolysis, and gluconeogenesis (11, 12). Conversely, a reduction in thyroid hormone levels, hypothyroidism, is associated with a hypo metabolic state characterized by reduced resting energy expenditure, weight gain, reduced lipolysis, and reduced gluconeogenesis (8). The issue of the pathways involved in the thyroid disrupting effect exerted by PFAS was recently reviewed (13). As stated by Ghassabian et al., any step in the biosynthesis and secretion of thyroid hormones could potentially be affected by PFAS exposure. Several mechanisms could be envisaged, including: i) impairment of iodine uptake by thyroid cells through a competitive mechanism and/or direct inhibition of the sodium/iodide symporter (NIS); ii) interference with thyroglobulin synthesis; iii) modification of Thyroperoxidase (TPO) activity; iv) interference with feedback mechanisms or with thyroid hormone biological effects through disruption of TH signaling pathway, deiodinase enzyme activity or TH binding proteins (13). Aim of this article is to review current data on the thyroid disrupting effect of PFAS, particularly focusing on the differences between long- and short-chain compounds, and on newly emerging PFAS. *In vitro* findings, data in exposed workers, and hazards for maternal-infant thyroid status will be reviewed.

### Long-Chain PFAS: PFOA and PFOS

Concern on possible risks of PFAS for human health emerged when long-chain perfluoroalkyl carboxylic acids (PFCAs) and long-chain perfluoroalkane sulfonic acids (PFSAs) were shown to be ubiquitously present in the human body (14, 15). The long-chain PFAS include perfluorooctane sulfonic acid (PFOS) and perfluorooctanoic acid (PFOA). For many decades PFOA and PFOS were used to give surfactant properties to a variety of industrial products. There is compelling evidence for their persistence in the human body for long time. PFOS belongs to the PFSAs, whereas PFOA belongs to the PFCAs. Originally, the PFOA exposure was associated with thyroid dysfunctions and with kidney and testicular cancer, while PFOS exposure was mainly linked to decreased fertility and adverse developmental effects in fetus. Exposure to PFOA and PFOS was also correlated to an impaired immune response in children and to increased cholesterol levels and obesity in adults (7). Due to these hazardous effects, long-chain PFAS and their precursors were phased out by major manufacturers in Europe, Japan and U.S. thanks to the U.S. Environmental Protection Agency (EPA) 2010/15 PFOA Stewardship Program (16). Consequently, alternative compounds were synthetized, which mainly include short-chain fluorotelomer-based products. These short-chain compounds were considered to be safer for human health and for the environment.

Time trends from National Health and Nutrition Examination Survey (NHANES) showed reduced serum levels of PFOA and PFOS since the phase-out of these compound, indicating a reduced human exposures (2). On the other hand, long-chain PFAS continue to be produced by those companies which did not agree with stewardship commitments. Thus, despite reduced levels, PFAS were still detectable in 95% of NHANES subjects in 2013–2014 (17). Consistent serum levels for PFOS (35.3 ng/ml) and PFOA (5.6 ng/ml) were also found in pregnant women (18). More than 60% of U.S. children (aged 3–11 years) who were born after PFOS and PFOA were phased out still had detectable levels of 14 PFAS, including PFOS and PFOA (19). The European Food Safety Authority (EFSA) recently reassessed the risks of these substances for human health after a request of the European commission by performing a second evaluation taking into account data emerged after the first survey in 2008. The CONTAM group (EFSA’s Panel on Contaminants in the Food Chain) is assessing possible risks to human health from PFAS other than PFOS and PFOA (20). On July 4, 2020, restrictions carried out by the European Chemicals Agency (ECHA) on PFOA came into force following further scientific assessments.

### Short-Chain and Emerging Replacement PFAS

With the phase-out of long-chain PFAS, companies shifted to short-chain fluorotelomer-based chemistry. These shorter PFAS are commonly known as C6 PFAS because they contain six fully fluorinated carbon groups with additional non-fluorinated carbons. By substituting long-chain PFAS with the short-chain ones, industry expected that the latter compounds might be both less toxic than the original PFAS and less persistent in the human body. However, data on the safety and bio-persistence of these shorter PFAS are still controversial (21, 22). Indeed short-chain PFASs seem to be persistent as their long-chain counterparts and show different, but not less worrisome effects on human health. Their widespread distribution in the environment is also a matter of concern (21, 23, 24). The main problem of these compounds is that they do not break down quickly after their intended use; consequently, they are not easily cleared up from the environment. In addition, the activated carbon filtration methodology, currently used to remove long-chain compounds from water, is less effective in removing the short-chain compounds (21). Short-chain PFAS may be divided in two main types: PFASs with carbon chain lengths of 5 and lower, and PFASs with carbon chain lengths of 7 and lower. Among these short-chain compounds, data on kinetics and toxicity in humans mainly regard perfluorohexanesulfonic acid (PFHxS). Its effects are similar to those of PFOS, making PFHxS a poor alternative. Although data regarding other short-chain PFAS are scanty, data from the literature support the hypothesis that they might be less toxic than PFOS and PFOA. Another short-chain PFAS, pentafluorobenzoic acid (PFBA) was found to be present at high concentrations in human tissues and especially in the brain. These data would imply that the short-chain PFAS might bio accumulate in humans at a higher degree than what expected from experiments in animals. Moving to environmental effects, it would seem that PFOS/PFOA and other long-chain PFAS are generally more toxic than the short-chain analogues even if the toxicity of short-chain PFAS is still not sufficiently investigated. However, some investigations indicate that at least the short-chain Fluorotelomer alcohols (FTOHs) may have higher endocrine disrupting effect than its analogue long-chain FTOHs (25).

Further alternatives to long-chain PFAS are represented by large fluoro-ether PFAS such as perfluoro-2-propoxypropanoic acid (GenX) and 4,8-dioxa-3H-perfluorononanoate (ADONA). These compounds were engineered with ether linkages and sites of hydrogenation in the effort to reduce their biological half-life (26). These PFAS replacements are produced in large quantities and recently, the public attention has shifted towards important levels of these PFAS compounds found from drinking water in North Carolina. One of these commercial mixtures was GenX, a DuPont’s substitute for PFOA. Data regarding the possible toxicity of these new emerging PFAS are scanty, but the few ones are not encouraging. Indeed, as shown in recent *in vitro* studies (27, 28), the toxicity of GenX was reported to be greater as compared with that of PFOA and PFOS. This is because GenX activates the Peroxisome-proliferator-activated receptor alpha (PPARα) pathway in mouse liver cell lines and causes cell injury (29). In animal models, GenX was also shown to be hepatotoxic and cancerogenic (28, 30–32). GenX is also highly mobile in the environment being detected faraway from its source. In consideration of the above reported data, GenX was marked as a Substance of Very High Concern (SVHC) by the ECHA in June 2019 (33). At difference with GenX, the exposure to another new generation PFAS named Perfluoro {acetic acid, 2-[(5-methoxy-1,3-dioxolan-4-yl)oxy]}, ammonium salt (C6O4) was recently demonstrated to not exert adverse effect on thyroid cells *in vitro* (34). **Table 1** shows the most representative PFAS divided in three main groups as Long-Chain, Short-chain and Emerging PFAS.

**Table 1:** The most representative PFAS were divided in three main groups as Long Chain, Short-chain and Emerging PFAS showing each chemical structure.

LONG CHAIN PFAS	
perfluorooctanoic acid (PFOA)	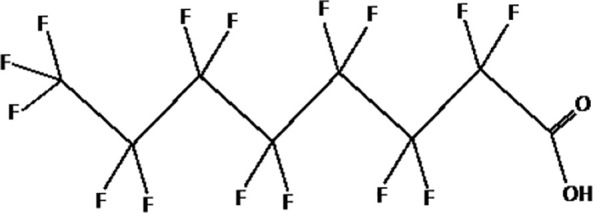
perfluorooctane sulfonic acid (PFOS)	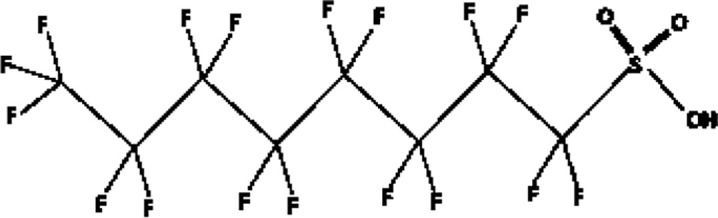
perfluorodecanoic acid (PFDeA)	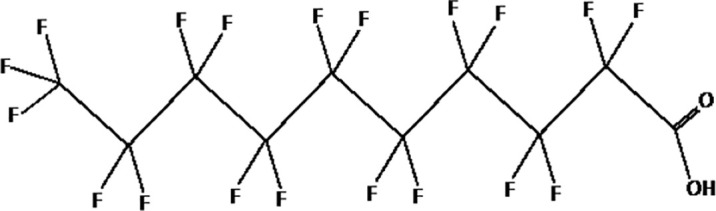
perfluoroundecanoic acid (PFUnDA)	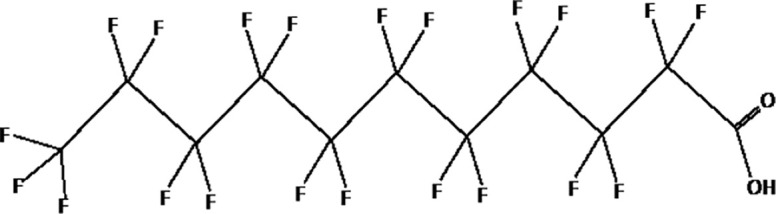
Perfluorododecanoic acid (PFDoDA)	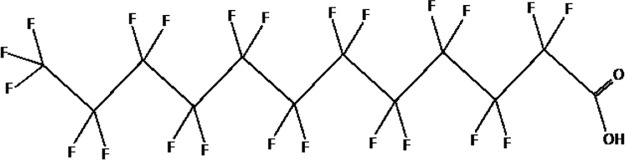
Perfluorotridecanoic acid (PFTrDA)	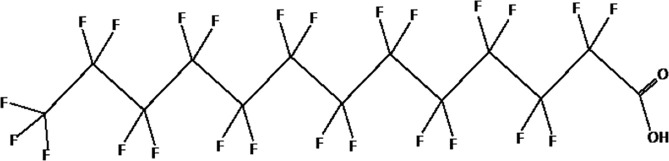
Perfluorotetradecanoic acid (PFTeDA)	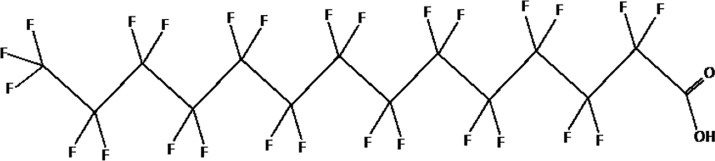
Perfluorooctanesulfonamide (FOSA)	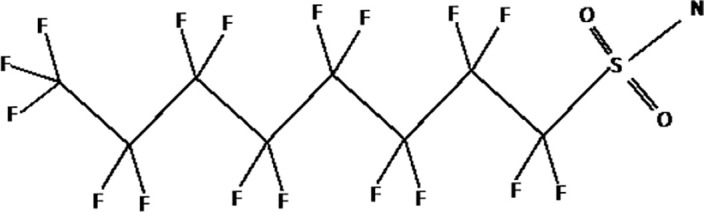
N-methyl-perfluorooctane sulfonamidoacetic acid (MeFOSAA)	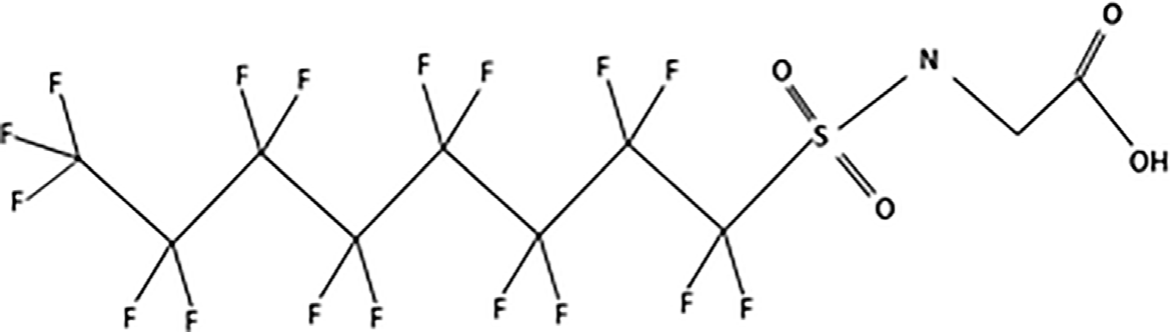
N-ethyl-perfluorooctane sulfonamidoaceticacid (EtFOSAA)	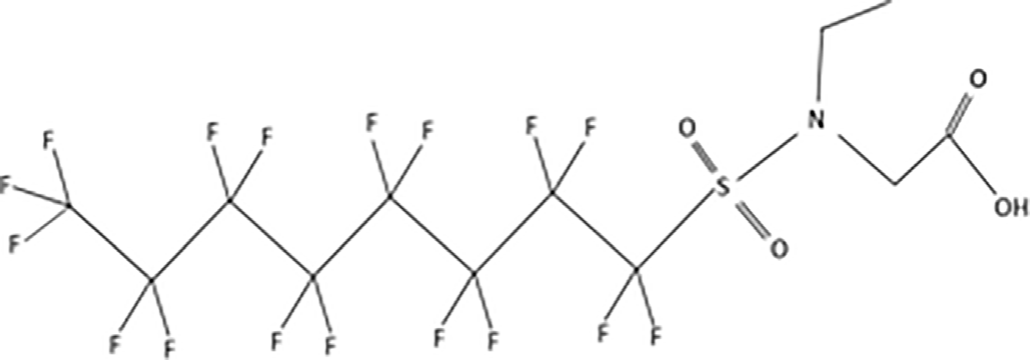
**SHORT CHAIN PFAS**	
perfluorohexane sulfonic acid (PFHxS)	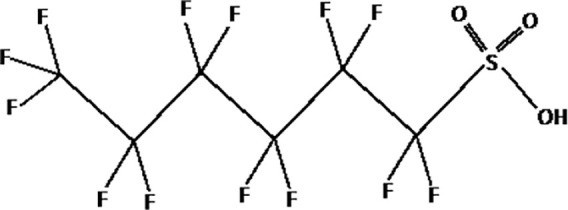
Pentafluorobenzoic acid (PFBA)	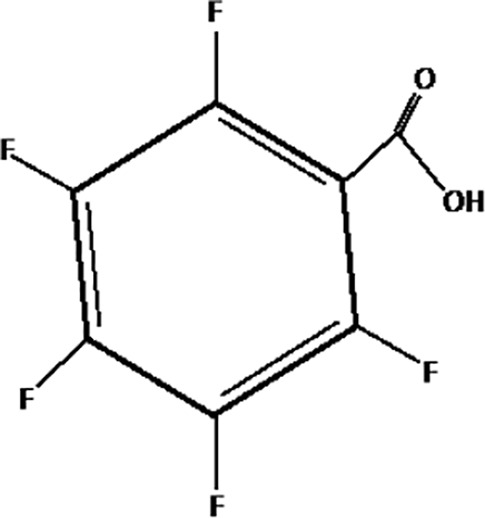
Perfluorobutane sulfonic acid (PFBS)	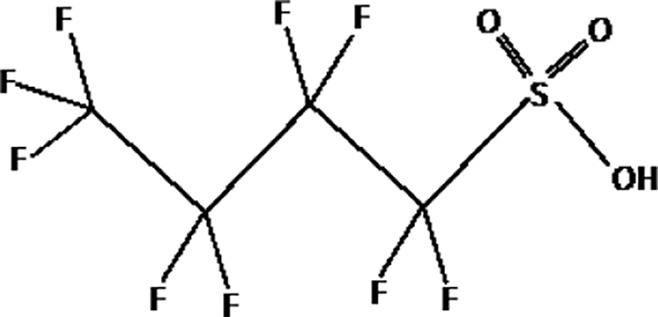
Undecafluorohexanoic acid (PFHxA)	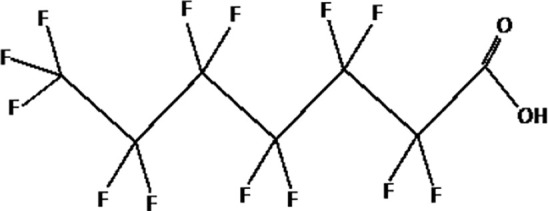
**EMERGING PFAS**	
Perfluoro-2-propoxypropanoic acid (GenX)	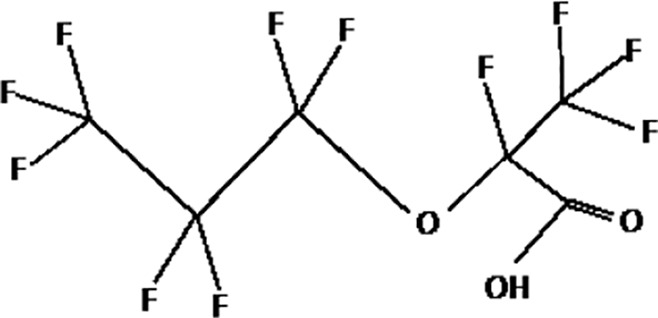
4,8-dioxa-3H-perfluorononanoate (ADONA)	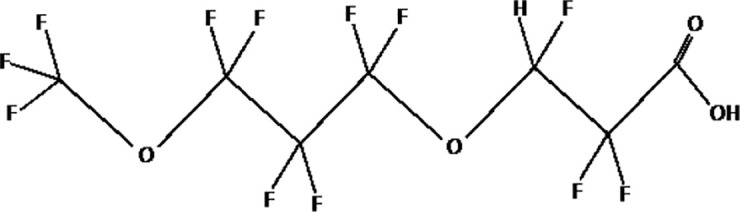
Perfluoro{acetic acid, 2-[(5-methoxy-1,3-dioxolan-4-yl)oxy]}, ammonium salt	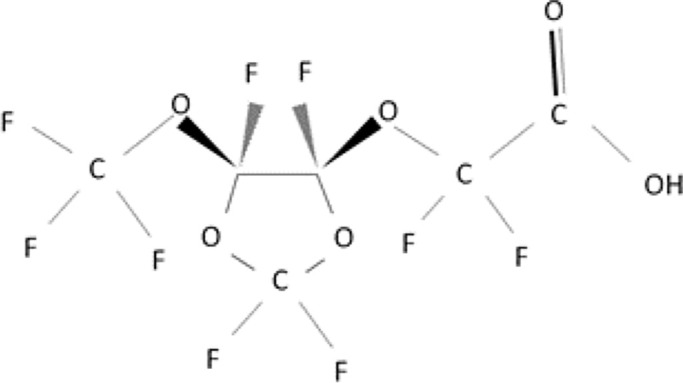

To summarize our current knowledge, long-, short-chain, and emerging PFAS are potentially dangerous for human health making further studies and monitoring mandatory.

### Endocrine Disruptive Effects of New and Old Generation PFAS on the Thyroid Gland

Endocrine-Disrupting chemicals (EDCs) are defined by the Endocrine Society as: “an exogenous [non-natural] chemical, or mixture of chemicals, that interferes with any aspect of hormone action.” Because of the endocrine system’s critical role in so many important biological and physiological functions, impairments in any component of the endocrine system can lead to disease (35, 36). Due to the thyroid gland and thyroid hormones important role in normal maintenance of metabolism and their critical role in fetal and childhood development, the disruption of thyroid function could cause several problems for the human health. Several studies recognized the ability of PFAS to interfere with hormone systems acting as EDCs (7, 35, 36). However, results were not consistently reported by all studies, being the discrepancy higher when a comparison among long-, short-, and new emerging PFAS were made. Nevertheless, PFAS potential interference with thyroid function was investigated taking into account different categories of subjects including exposed workers, general population, and pregnant women and infants. In addition, *in vitro* experiences might be also helpful for understanding the mechanisms of actions of these compounds.

### *In Vitro* Experiences

Laboratory studies on thyroid cell cultures showed that *in vitro* exposure of thyroid cells to several PFAS can have various thyroid-disrupting effects. An accumulation of PFOS and PFOA was documented in thyroid cells, and a cytotoxic effect was observed after exposure to extremely high concentrations of these compounds (37). In addition, PFOS and PFOA reduce thyroperoxidase (TPO) activity in tumor thyroid cells lines (38) and PFOS was suggested to act as a thyroid hormone receptor (THR) agonist in GH3 cells as found by using THRα and THRβ-mediated luciferase reporter assay (39). Interestingly, Conti et al., showed that PFOS (but not PFOA) is able to inhibit the iodide accumulation in FRTL-5 cells mediated by NIS. The same finding was reported in non-thyroid cells with an eterologus NIS expression (40).

A recent *in vitro* study compared the potential thyroid disrupting effects of long-short-chain PFAS (PFOA and PFOS) and four different short-chain PFAS [including perfluorobutane sulfonic acid (PFBS), PFBA, perfluoroalkyl phosphonic acid (PFPA), and perfluoropentanoic acid (PFPeA)]. The results showed that short-chain PFAS had no cytotoxic effects on rat thyroid cells and did not interfere with thyroid-stimulating hormone (TSH)-dependent cyclic adenosine monophosphate (cAMP) production (41).

Regarding new emerging PFAS, a recent study by Coperchini et al., reported that FRTL-5 cells exposed to increasing concentrations of GenX displayed both genotoxic and cytotoxic effects (42).

### Data on Animal Models

Evidences derived from animal models suggested that PFASs may alter thyroid hormone homeostasis (7), however, given the toxic kinetics and tissue distribution of PFASs that is known to be species-specific (2), extrapolating data from animals studies and compare them to humans does not allow to draw firm conclusions.

Previous data obtained in laboratories mainly found that PFAS reduce the circulating levels of thyroid hormones in diet-exposed animals, mainly by increasing their metabolic clearance rate (43). The thyroid disruptive effect of PFAS was investigated in recent studies. In particular, two main studies investigated the effect of PFOA and the new emerging PFAS, GenX, in mice and rats. Conley et al., characterized the potential maternal and post-natal toxicities of oral GenX in Sprague-Dawley rats during sexual differentiation and assayed circulating maternal thyroid hormones (44). They found that GenX caused a dose-responsive up-regulation of 28 different genes involved in the PPAR signaling pathway (influencing lipid catabolism, fatty acid uptake, and lipoprotein transport and assembly. The majority of shared, up-regulated genes were associated with fatty acid metabolism. A similar effect was found in pregnant mice livers. Maternal serum displayed dose-responsive decrease in all measured thyroid hormones, indeed total thyroxine (T4) levels were significantly lower than non-exposed pregnant mice, while serum total triiodothyronine (T3) levels were below assay detection levels (i.e., <0:2 ng=ml), at least for higher doses. These data are in line with previous studies in mice which found that PFOS and PFOA reduced both T3 and T4 in mother serum. The only difference between these studies was related to the fact that in the earlier ones T4 rather than T3 was reduced at a greater extent (44). Another study in mice is the one by Blake et al., who compared the toxicity of PFOA and GenX in pregnant mice. They reported that in mice exposed to PFOA or GenX, high levels of both compounds could be retrieved in the placenta. In addition they found an increase in placenta weight, an increase in placental lesions, and lower embryo-placenta weight ratios. A placental thyroid hormones disruption was specifically exerted by GenX (31).

Zebrafish represents another well established model for studying the effects of PFAS, and recent studies provided potentially interesting findings. Indeed, Zhang et al., recently reported increased whole-body thyroid hormones in zebrafish larvae [embryo collected at 0.5 to 2.0 h post fecondation (Hpf). Exposure ended 96 hpf)] exposed to 8:8 PFPiA, Perfluoroalkyl phosphinic acids (PFPiAs) a class of per- and PFAS along with perfluoroalkyl phosphonic acids (PFPAs). In details, after exposure to 0.5–50 nM of 8:8 PFPiA in zebrafish larvae, an increase of T4 and T3 was observed. In addition, corticotropin-releasing hormone (CRH) and TSHβ were down-regulated and uridinediphosphate-glucuronosyltransferase (UGT1AB) resulted up-regulated. The authors suggested that this should be regarded as a compensatory response to the hyperthyroid status (45). On the contrary, the recent study by Kim et al., showed an up-regulation of corticotropin releasing hormone b (CRHB), thyrotropin receptor (TSHR), and thyroid transcription factor-1 (NKX2.1) genes which was suggestive of a negative feedback in response to decreased circulating thyroid hormones (46). Finally, Rodriguez-Jorquera et al., performed a blood transcriptomic analysis in fish exposed to PFAS reporting an up-regulation, mainly promoted by PFOS, of thyroid hormone receptor beta (TR-β) (47). This increase expression was hypothesized to be involved in a non-correct thyroid hormones binding and consequent alteration of the hypothalamus-pituitary-thyroid axis function. This study highlighted that also small concentrations of organic contaminants may be detected in the bloodstream (47).

Another excellent model to test thyroid axis disruption is *Xenopus laevis*; indeed, thyroid hormones are highly conserved across vertebrates (48). In a recent study, Fini et al., investigated the potential consequences of human amniotic fluid exposure to some chemical contaminants during embryonic development on thyroid hormone signaling (49). Exposure to a mixture of chemicals including PFOA and PFOS at those concentrations found in human amniotic fluid affected thyroid hormone-dependent transcription, gene expression, brain development, and behavior in early embryogenesis. In particular a dose-dependent effect of short-term (72 h) exposure to PFOA and PFOS alone was observed. These results suggested a potential adverse effect of PFAS exposure and fetal development (49).

The potential adverse role of PFAS for thyroid function was also investigated in cats. In particular, the interesting study by Wang et al., compared the ΣPFAS levels in the serum of Northern California cats and humans collected during two time periods: 2008 to 2010 and 2012 to 2013 (50). The ΣPFAS was composed of: PFOS, PFOA, perfluorohexane sulfonate (PFHxS), perfluorononanoic acid (PFNA), perfluorodecanoic acid (PFDeA), perfluoroundecanoic acid (PFUnDA), N-methyl-perfluorooctane sulfonamidoacetic acid (MeFOSAA) had detection frequencies, Perfluorooctanesulfonamide (FOSA) and N-ethyl-perfluorooctane sulfonamidoaceticacid (EtFOSAA). The results showed that circulating concentrations of long-chain perfluorinated carboxylic acids, especially PFNA and PFUnDA, were significantly higher in cat than in humans. Furthermore, serum from hyperthyroid cats in the second time period showed higher ΣPFAS level (9.50 ng/ml) compared to non-hyperthyroid cats (7.24 ng/ml). In particular, serum PFOS levels were significantly higher in the hyperthyroid cats. This result may indicate a possible link between PFAS levels and hyperthyroidism in cats (50). Moving on data on great mammalians, Routti et al., examined the correlation between levels of PFAS and thyroid hormones and genes transcripts in in adult male Atlantic walruses (n ¼ 38) from Svalbard (Norway). They found that transcript levels of nuclear receptor subfamily 3 group C member 1 (NR3C1), the thyroid hormone receptor alpha (THRα) and the retinoic X receptor alpha (RXRα) were negatively correlated with plasma ΣPFAS concentrations. However, these results should be interpreted with care since statistical significance was reached only when three outliers were excluded (51). Very limited data are available regarding the possible effect of chronic exposure to PFAS on thyroid morphology, coming from experimental animal models. Indeed, it was demonstrated by using a zebrafish model that chronic exposure to PFOS would alter the normal structure of thyroid follicular cells (52). Chen et al. reported a significant reduction of the nuclear area of thyroid follicular epithelial cells of fishes chronically exposed to PFOS. Moreover, mitochondria and endoplasmic reticulum of zebrafish chronically exposed to PFOS were significant altered as evidenced by Transmission electron microscopy analysis showing structural modifications in intracellular organuli, such as ribosomes, mitochondria, and endoplasmic reticulum. At the tissue level, chronic PFOS exposure also caused oedema of the interstitial tissue. These morphological changes in the structure of thyroid gland were accompanied by disturbances in thyroid function, as assessed by a decline in circulating thyroid hormone concentrations in this animal model (53).

On the other hand, a study performed on dams exposed to PFHxS reported no statistically significant effects on thyroid gland weights and histopathology (54).

Taken together the above reported laboratory evidences highlight the adverse effects of the new emerging PFAS on thyroid hormones and thyroid-related-genes suggesting that they could also be more dangerous than their predecessors long-chain PFAS.

### Interference of PFAS on Thyroid Function: New Data Collected From Exposed Workers, General Population, and From Patients With Underlying Thyroid Diseases

Studies performed before 2017 regarding the interference of PFAS with thyroid function in humans, mainly highlighted a higher occurrence of hypothyroidism due to the exposure to PFOA (and even if with fewer evidences to PFOS) both in the general population and in the exposed communities (7). In 2018, a study performed in Korean adult general population, found that the 10-year trend of circulating levels of 13 PFAS gradually increased from 2006 to 2013 and subsequently decreased over the following years. In particular, it was found a significant correlation between free thyroxine (FT4) levels and PFNA, PFHxS, and PFDA, moreover significant differences were observed between participants with and without diabetes as to the levels of PFHxS and PFDoDA. In particular, some gender-related specificities were found. Indeed, while in male patients, PFNA, PFOS, PFDA, and PFDS showed the strongest positive correlation with serum FT4 levels, in females FT4 levels were more strongly related to PFBS, PFHxA, PFHxS, PFNA, PFOS, PFDA, and PFUnDA (20). Taken together, positive associations between FT4 and PFOS, PFNA, PFHxS, PFDA, and PFOA levels were registered pointing out a stronger correlation of PFOS with thyroid hormone levels than PFOA, confirming results of previous studies (55–57). Finally, in both male and female participants, TSH showed no significant correlations with PFAS serum levels. The positive correlation observed within PFAS and thyroid hormone levels was attributed to the interference of these compounds with the binding of FT4 carrier proteins which ultimately would disrupt thyroid hormone homeostasis and affect basal metabolic rate and protein synthesis (58). Another study longitudinally evaluated the PFAS circulating levels within and between individuals over nearly two decades as well as their relationship to thyroid hormones levels recruiting in this case participants who lived or worked within a 5-mile radius of a Feed Materials Production Center collected as early as 1991 in the Fernald Community Cohort (59). The study investigated the relationship between changes in PFAS serum levels and repeated measures of total T4 and TSH, reporting significant positive associations between PFOS and an increase in circulating TSH levels. On the other hand, no associations between PFAS and total T4 or TSH were detected by using crude models of latent effects. In the adjusted latent model, a positive association between serum PFNA and total T4 was found. Finally, by using the adjusted latent model [which included covariates for age, year of measurement, sex, education, income, marital status, and body mass index (BMI)] an increase in total T4 was related to serum PFNA levels, with no relationship with TSH (59). A meta-analysis performed by Kim et al. (which considered all studies from 1985 to 30 April 2017 regarding serum levels of PFOA, PFOS, and PFHxS) reported that blood PFOS concentrations were positively correlated with free T4 and negatively correlated with total T4 and total T3. PFOA concentrations were negatively correlated with total T4 while PFHxS showed a negative correlation with total T4, finally suggesting that PFASs would negatively associate with total T4 (60). In a study evaluating the associations between serum PFAS concentrations and circulating thyroid hormones in a population of Alaska natives it was suggested that the effects of PFAS exposure on thyroid hormone homeostasis may differ between sexes. Indeed, PFOS and PFNA were positively associated with circulating free triiodothyronine (FT3) in females and negatively associated with FT3 in males participants (61).

In a general population study with long-term high exposure to PFHxS, PFOS, and to a lesser extent PFOA through drinking water constituted by an open (dynamic) cohort, defined as people who have lived in Ronneby municipality at any time during the time period 1980–2013, no increase in the risk of clinically detected hyperthyroidism was observed, neither in men nor in women (62).

## Prenatal Exposure to PFAS: Effects on Maternal and Neonatal Thyroid Function

Neonatal and maternal thyroid function are critical for children growth and neurodevelopment. Indeed, during early pregnancy, the fetus is dependent on maternal thyroid hormones until thyroid hormone fetal production increases around 18–20 weeks of gestation (63). Also after birth a normal thyroid function is required for a regular neurodevelopment and growth (64). Alterations in maternal thyroid function during pregnancy are associated with several adverse fetal outcomes, such as neurodevelopmental deficits, preterm delivery, and reduced fetal growth (65). Additionally, neonatal thyroid dysfunction has been associated with impaired neurodevelopment (66–68). Many epidemiological studies investigated the relationship between exposure to PFAS during the prenatal period and alterations in maternal and neonatal thyroid function. In particular, many studies published prior to the dismissing of PFOA and PFOS indicated that exposure to several long-chain PFAS could influence TSH and thyroid hormone levels both in mothers and newborns. Although results were not completely consistent, an overall association between higher PFAS serum/plasma levels and a reduction in T4 levels and/or an increase in TSH levels was described (69), especially for PFOS. The effect on circulating TSH and thyroid hormones was generally small and likely of limited clinical significance, even if available evidences suggested that women with autoimmune thyroiditis might be at higher risk for developing subclinical hypothyroidism during pregnancy if exposed to PFAS (7).

Several clinical studies have been published in the last three years regarding the possible relationship between PFAS exposure and thyroid dysfunction during pregnancy. These studies are particularly relevant since they belong to the post-out of production era of long-chain PFAS, even if, due to their long half-life, persistency, and bioaccumulation these compounds can still be found in patient’s sera, although at lower concentrations. In the last years important data derived from several prospective large series maternal cohorts. The results of these studies are summarized in **Table 2**. A specific focus on fetal outcomes of PFAS exposure and the possible role of « mixture » exposure to multiple PFAS emerged. Moreover, the possible role of thyroid autoimmunity in the mother and also the role of fetal sex were both considered as modifying factors.

**Table 2:** Summary regarding recent data from several prospective large series maternal cohorts.

Author	Year	Study Design	Clinical Setting	PFAS Exposure Evaluation	Evaluated Outcome	Results
Preston et al. (70)	2018	Prospective cohort study	732 mothers and 480 newborns belonging to a longitudinal prebirth cohort in Boston, Massachusetts (Project Viva).	Early pregnancy (median 9.6 wk gestation) maternal plasmatic levels of PFOA, PFOS, PFNA, PFHxS, EtFOSAA and MeFOSAA	Maternal plasma levels of TSH, total T4, T3 resin uptake, free T4 index and TPO Ab. Neonatal total T4 plasma levels from heel sticks	Maternal PFOA, PFHxS,and MeFOSAA concentrations are inversely related with maternal free T4 index. PFOA, PFOS, and PFNA concentrations are inversely related with TSH levels only in TPOAb positive women. Prenatal PFOS, PFOA,and PFHxS maternal concentrations are inversely related with T4 levels only in male, but not in female neonates.
Preston et al. (71)	2020	Prospective cohort study	726 mothers and 425 neonates belonging to a longitudinal prebirth cohort in Boston, Massachusetts (Project Viva).	Early pregnancy (median 9.6 wk gestation) maternal plasmatic levels of PFOA, PFOS, PFNA, PFHxS, EtFOSAA and MeFOSAA. Both individual and joint effects of PFAS exposure evaluation	Maternal plasma levels of TSH, total T4, T3 resin uptake, free T4 index, and TPO Ab. Neonatal total T4 plasma levels from heel sticks	The mixture of the six PFAS is associated with significantly lower maternal Free T4 index, with PFOA, MeFOSAA, EtFOSAA and PFHxS contributing most to the overall effect. In infants, higher concentrations of the PFAS mixture are associated with lower T4 levels, especially in males, with PFHxS and MeFOSAA giving the highest contribution to this effect.
Inoue et al. (72)	2019	A cross-sectional analysis	1366 maternal blood samples from women enrolled in the Danish National Birth Cohort (DNBC) between 1996 and 2002.	PFOS, PFOA, PFHxS, PFHpS, PFNA and PFDA maternal serum levels.	Percentage changes of maternal serum TSH and fT4 levels collected between the 5th and the 19th gestational week according to gestational-week specific ranges	Adjusted TSH values were higher among women with the PFOS, PFOA, PFHxS, and PFHpS levels in the highest quartile when compared with the lower quartiles, only in samples collected between the 5th and the 10th gestational week, but the difference became null after 10th gestational week. Women with higher PFDA levels had higher fT4 levels before the 10th gestational week
Liew et al. (73)	2020	Nested case-control study	220 pregnancies ending in miscarriage during weeks 12–22 of gestation vs 218 pregnancies resulting in live births taken from the Danish National Birth Cohort (DNBC) between 1996 and 2002.	PFOS, PFOA, PFHxS, PFHpS, PFNA, and PFDA maternal serum levels.	Odds ratios for miscarriage (adjusted for maternal age, parity, socio-occupational status, smoking, alcohol intake, and maternal history of miscarriage)	Higher odds ratios for miscarriage in women with PFOA, PFOS, and PFHpS in the highest quartiles, with a greater effect in parous women
Aimuzi et al. (74)	2020	Prospective observational study	1885 pregnant women enrolled in the Shanghai Birth Cohort	PFBS, PFOA, PFHpA, PFOS, PFHxS, PFNA, PFUA, PFDA, PFDoA and PFOSA maternal serum levels collected before the 16th gestational week	Maternal TSH, FT3, FT4, and TPOAb (adjusted for pre-pregnancy BMI, maternal education, gestational age, maternal age, fish intake)	PFOA and PFHxS are directly associated with FT4 levels, PFNA and PFHxS are directly associated with FT3 levels. PFHxS is inversely related with TSH levels. The entity of all associations is greater in TPO Ab-positive women.
Itoh et al. (75)	2019	Prospective cohort study	701 mother−neonate pairs recruited in a prospective birth cohort (the Hokkaido Study) between 2002 and 2005	PFHxA, PFHpA, PFOA, PFNA, PFDA, PFUnDA, PFDoDA, PFTrDA and PFTeDA	TSH, FT3, FT4, TgAb and TPOAb in maternal early-pregnancy samples and in cord blood samples (age at delivery, parity, pre-pregnancy BMI, educational level, alcohol consumption and smoking habit during pregnancy)	In thyroid-antibody negative women maternal FT3 levels are positively associated with maternal PFHxS. In thyroid-antibody positive women, PFNA is positively related with maternal FT3, PFOA is inversely related with maternal TPOAb titer. In male newborns, maternal PFOS levels are directly related with TSH levels of the newborn in children from thyroid-antibody negative mothers but not in those from thyroid-antibodiey positive mothers. In male newborns from TA-negative mothers, PFDA and PFUnDA are inversely associated with the newborn FT3 levels. In male newborns from TA-positive women, maternal PFDA is inversely associated with TSH levels of the children. In female newborns from TA-negative mothers, PFDoDA maternal levels are directly related with the girls’ TSH levels; PFDA and PFTrDA levels are positively related with the girls FT3 levels. In girls’ from TA positive mothers PFDoDA maternal levels are inversely related with the girls FT4 levels. A direct relationship between PFOA, PFNA, and PFDA levels and TgAb levels is observed in girls born from TA positive women.
Lebeaux et al. (76)	2020	Prospective cohort study	468 pregnant women and their children from the Health Outcomes and Measures of the Environment (HOME) Study (Cincinnati, USA)	PFOA, PFOS,PFNA, and PFHxS in maternal and cord blood at 16th gestational week, 26 th gestational week and at delivery	TSH, FT3, FT4, TT4, and TT3 in maternal and serum at 16th gestational week and in cord blood at delivery	PFAS, considered individually or as mixtures, are not associated with any thyroid hormone parameter in the whole group. In multivariable models, PFOA, PFOS and PFHxS maternal levels are associated with a decrease in cord blood FT4 (but only among children born to mothers with positive TPOAb.
Reardon et al. (77)	2019	Prospective cohort study	494 women from a Canadian birth cohort (the APrON Study)	PFHpA, PFOA, PFNA, PFDA, PFUnA, PFDoA PFHxS, PFOS, and six different PFOS isomers	FT3,FT4, TSH and TPOAb maternal plasma levels collected in each trimester and 3-months postpartum.	Throughout pregnancy and post-partum, PFHxS levels are inversely associated with FT4 levels and directly associated with TSH levels. When only TPOAb-positive women are taken into account, negative associations are found between PFUnA levels and FT4 and between PFOS and TSH.
Xiao et al. (78)	2020	population-based prospective cohort study	172 mother-singleton pairs from the Faroe Islands.	PFHpA, PFOA, PFNA, PFDA, PFUnDA, PFDoDA, PFHxS, PFHpS, PFOS, and 3 PFAS precursors (NEtFOSAA, NMeFOSAA and FOSA) measured in maternal serum at 34th gestational week and in cord blood	Maternal and cord blood TSH, FT3 and FT4 levels, newborn birth weight, birth length, and cranial circumference adjusted for sex of the fetus, gestational age, maternal education, maternal pre-pregnancy BMI, parity smoke and alcohol consumption, and exposures to mercury or PCB	Inverse correlation between PFHxS, PFOS, PFNA, PFDA, and PFOA maternal levels and birth weight, birth length or cranial circumference. Direct correlation between PFNA, PFOS, PFDA, and PFOA levels with maternal TSH. Direct correlation between maternal PFOS, PFOA, PFDA, and PFNA and TSH cord blood levels. The relationship between PFAs and birth size is not mediated by thyroid function parameters
Luo et al. (79)	2020	Prospective cohort study	758 mother-child pairs from the Danish National Birth Cohort between 1996 and 2002.	PFOS, PFOA, PFHxS, PFHpS, PFNA, and PFDA serum levels at 8^th^ gestational week	Child’s behavioral difficulties using Strengths and Difficulties Questionnaire (SDQ) reported by parents (7 and 11 years) and children (11 years). Adjusted for maternal age, parity, socioeconomic status, prepregnancy BMI, gestational week of blood collection, smoking during the 1st trimester , alcohol intake during the 1st trimester, and parent’s behavioral scores.	Significant association between PFNA levels and child behavoural difficulties at 7 and 11 years, no consistent associations for other types of PFAS. Indirect mediating effects via maternal thyroid disfunction observed for PFOS, PFOA, PFNA, and PFHpS but only for parent-reported total, and externalizing behaviors at 7 years

Some of these studies focused directly on the effect of several PFAS on maternal thyroid function. For example, Inoue et al. (72) evaluated the gestational-week specific associations between maternal TSH and FT4 levels and the plasma concentrations of six different long-chain PFAS [PFOS, PFOA, PFHxS, perfluoroheptanesulfonic acid (PFHpS), PFNA, and PFDA] during the first and the second trimester of pregnancy. Samples were taken from a national Danish cohort of pregnant women enrolled between 1996 and 2002. The results showed that, even though in all women, the typical U-shaped trend of TSH levels modification was observed during the first two trimesters of pregnancy, higher levels of exposure to PFAS (in particular to PFOS, PFOA, PFHxS, and PFHpS) were associated with higher TSH levels when compared with women at lower levels of exposure. This significant difference could not be observed after the 10th week of gestation. The authors concluded that some gestational-week–specific association between high levels of exposure to several PFAS and TSH levels in early gestation could exist.

Another study aimed at evaluating the relationship between PFAS and maternal thyroid function parameters was the one by Aiumzi et al. (74) who performed a large prospective study on a cohort of 1,885 pregnant women included in the Shanghai Birth Cohort. In details, 10 different long-chain PFAS [including PFBS, PFOA, PFHpA, PFOS, PFHxS, PFNA, PFUNDA, PFDA, PFDoA, and Perfluorooctanesulfonamide (PFOSA)] were measured in maternal blood collected before the 16th gestational week and their levels were related with those of maternal TSH, FT3, FT4, and TPOAb. The results showed that PFOA and PFHxS were positively associated with FT4, while PFNA and PFHxS were positively associated with FT3. PFHxS was also negatively associated with TSH levels. The authors also separately analyzed results in TPO Ab positive and negative women. Among AbTPO negative women only a weak positive correlation between PFHxS and FT3 levels and an inverse U shaped relationship between PFHxS and FT4 was observed. In contrast, when only the TPO Ab positive women were taken into account, a significant direct correlation between PFNA and FT4 and significant, U-shaped, correlations between PFUNDA, PFOS, PFNA, and PFHxS with TSH levels was observed. These interactions appeared to be stronger in AbTPO women, as testified by higher beta values.

A very recent longitudinal study by Reardon et al. (77) extended the evaluation of a possible relationship between thyroid hormones and maternal thyroid function also to the postpartum period. The participants of a prospective Canadian birth cohort (the APrON Study), were enrolled, and data regarding FT3, FT4, TSH, and TPOAb maternal plasma levels were collected in each trimester and 3-months postpartum. The authors related thyroid function parameters and antibody levels to those of eight different PFAS (PFHpA, PFOA, PFNA, PFDA, PFUnDA, PFDoA PFHxS, and PFOS) as well as with the levels of six different isomers of PFOS. The results showed that PFHxS levels as well as the total branched isomers of PFOS were positively associated with TSH in mixed-effect models, with strongest associations in the early phase of gestation. Throughout pregnancy and post-partum, PFHxS levels were inversely associated with FT4 levels and directly associated with TSH levels. When only TPOAb-positive women were taken into account, negative associations were found between PFUnDA levels and FT4 and between PFOS and TSH. These data would support the notion that the presence of thyroid autoimmunity can influence the effect of PFAS on thyroid homeostasis throughout pregnancy and post-partum.

Although several data regarding the effects of PFAS on thyroid homeostasis in pregnancy are available from previous studies, more recent papers have added new information on this topic. Some studies have evaluated if maternal PFAS exposure could alter thyroid function parameters in the newborns.

Preston et al. (70) investigated the association between PFAS exposure during early pregnancy and maternal and fetal thyroid function in a prospective cohort study in Boston, Massachusetts. In detail, the plasma concentrations of six long-chain PFAS (PFOA, PFOS, PFNA, PFHxS, EtFOSAA, and MeFOSAA) were evaluated in early pregnancy (median 9.6 weeks of gestation). Thyroid function parameters were measured in the same plasma samples and included TSH, total T4, T3 resin uptake (an indirect measure of thyroid hormone binding protein saturation by thyroxine), free T4 index (calculated as total T4 × T3 Uptake) (FTI), and TPO Ab. Neonatal total T4 was obtained by whole blood on filter paper from heel sticks.

The results showed that, although PFAS concentrations were not associated with maternal T4, PFOA, PFHxS, and MeFOSAA concentrations were inversely associated with maternal free T4 index. PFOA, PFOS, and PFNA concentrations were inversely associated with TSH levels only in TPOAb positive women. Prenatal PFOS, PFOA, and PFHxS maternal concentrations were inversely associated with T4 levels only in male, but not in female neonates. The authors suggested that these results support a role for PFAS exposure in influencing thyroid function in both mothers and infants.

A more recent work by the same group (71) evaluated the association of exposure to a combination of the same PFAS, instead of the single ones, on maternal and fetal thyroid function. The results showed that higher concentrations of the PFAS mixture were associated with significantly lower maternal free T4 index, with PFOA, MeFOSAA, EtFOSAA, and PFHxS contributing most to the overall effect of the mixture. In infants, higher concentrations of the PFAS mixture were associated with lower T4 levels, especially in males, with PFHxS and MeFOSAA giving the highest contribution to this effect. The authors concluded that there may be combined effects of prenatal exposure to multiple PFAS on maternal and neonatal thyroid function, but the direction and magnitude of these effects may vary across individual PFAS.

Itoh et al. (75) evaluated the correlation between prenatal exposure to eleven different PFASs [PFHxA, PFHpA, PFOA, PFNA, PFDA, PFUnDA, PFDoDA, perfluorotridecanoic acid (PFTrDA), and PFTeDA] and the levels of thyroid function parameters and thyroid autoantibodies [Ab thyroglobulin (Tg) and AbTPO] both in maternal and in cord blood. A multivariate linear model was constructed entering age at delivery, parity, pre-pregnancy BMI, educational level, alcohol consumption, and smoking habit during pregnancy as covariates. The main outcome was that both the newborn sex and the presence or absence of thyroid autoantibody in the mother could modify the relationship between maternal PFAS exposure and thyroid function.

Indeed, in thyroid-antibody negative women, maternal FT3 levels were positively associated with PFHxS, while in thyroid-antibody positive women PFNA plasma levels were positively related with FT3 levels. PFOA levels exhibited instead a significant inverse association with maternal TPOAb titer. No significant association between any PFAS and FT4 or TSH levels were observed independently from thyroid Abs status.

Next, the authors investigated the relationship between maternal PFAS exposure and the newborn thyroid function and thyroid autoantibody levels, in relation to the sex of the newborn. In male newborns, maternal PFOS levels were directly related with the TSH levels of the newborn in children from thyroid-antibody negative mothers but not in those from thyroid-antibody positive mothers. Always in male newborns from TA-negative mothers, PFDA and PFUnDA were inversely associated with the newborn FT3 levels.

At difference, in male newborns from TA-positive women, maternal PFDA was inversely associated with circulating TSH levels of the children.

On the other hand, in female newborn from TA-negative mothers, PFDoDA maternal levels were directly related with the girls TSH levels and PFDA and PFTrDA levels were positively related with the girls FT3 levels. Instead, in girls from TA positive mothers, an inverse relationship between PFDoDA maternal levels and the girls FT4 levels was observed. Lastly, a possible relationship between PFAS maternal levels and thyroid autoantibody levels in the offspring was sought. A direct relationship between PFOA, PFNA, and PFDA levels and TgAb levels was observed in girls born from TA positive women. The authors concluded that the relationship between different PFAS exposure and thyroid function perturbation in mothers and newborn is a complex phenomenon, likely influenced by both maternal autoimmunity and fetal sex.

The sex-based differences in the thyroid disrupting effects of PFAS exposure during pregnancy is intriguing, but the underlying mechanisms are still far from being elucidated. At present two main hypothesis were formulated. Itoh et al., suggested that differences in the elimination kinetics of PFAS between males and females, as previously shown for PFOA and PFOS in animal and in human studies may account for the different effects (80, 81), while other authors suggested that estrogen-induced increase in Thyroxine-binding globulin (TBG) production could alter measurements of circulating thyroid function parameters in newborn girls (82). The potential effect of maternal autoimmunity was also addressed by Lebeaux et al. (76) in a study examining data coming from a prospective cohort, the Health Outcomes and Measures of the Environment (HOME) Study, in which 468 pregnant women and their children coming from the Cincinnati area in US were enrolled. In particular the authors assessed the possible associations between TSH, FT3, FT4, TT4, and TT3 in maternal and cord blood and levels of four different PFAS (PFOA, PFOS, PFNA, and PFHxS) in the same samples. Two mixture modeling approaches [Bayesian kernel machine regression (BKMR) and quantile g-computation] and a multivariate linear regression were employed. The results showed that the levels of most of the analyzed PFAS were not associated with any thyroid function parameter, neither when considered alone nor as mixtures. When only women with positive thyroid autoantibodies were taken into account, a significant, even if slight, association between PFOS, PFOA, and PFHxS levels and fetal FT4 could be evidenced.

Only few studies evaluated whether the possible effect of PFAS on thyroid function could, in the end, result in clinically evident events, such as pregnancy complications or alterations in pregnancy development.

A very recent paper from the same Danish group of the study by Preston et al. (73). describes the results of a nested case-control study performed on the same aforementioned national cohort of pregnant women. In this case, the plasma levels of the six PFAS were compared between a group of 220 women who experienced miscarriage during weeks 12–22 of gestation and a group of 218 women with pregnancies resulting in live births. Data were adjusted for maternal age, parity, socio-occupational status, smoking, alcohol intake, and maternal history of miscarriage. The authors observed an increase in odds for miscarriage associated with increasing PFOA and PFHpS levels. In particular, the odd ratios (ORs) for having a miscarriage comparing the highest PFOA or PFHpS quartile to the lowest were 2.2 and 1.8, respectively. The ORs were also elevated for the second or third quartile of PFHxS or PFOS, but no consistent exposure-outcome pattern emerged. This association was stronger in parous women, while findings were inconsistent among nulliparous women.

Another very recent paper by Xiao et al. (78). studied a population-based prospective cohort of 172 mother-singleton pairs from the Faroe Islands, a population known to consume high quantities of whale meat, which implies elevated PFAS intake. In detail, the maternal serum levels of nine different PFAS (PFHpA, PFOA, PFNA, PFDA, PFUnDA, PFDoDA, PFHxS, PFHpS, PFOS) as well as three different PFAS precursors (NEtFOSAA, NMeFOSAA, and FOSA) were obtained at 34 weeks of gestation. In parallel, thyroid function parameters were evaluated in both cord and maternal serum. The results showed an inverse correlation between neonatal outcomes and PFAS levels. In particular, a doubling of PFHxS and PFOS levels was associated with significant reduction in birth weight, birth length, and head circumference. A less relevant effect was also observed for other PFAS: an inverse associations between PFNA, PFDA, and PFOA with birth weight and between PFDA and PFHpS with birth length was found, with similar effects in boys and girls. Higher PFNA, PFOS, PFDA, and PFOA maternal levels were associated with an increase in maternal TSH levels. When the correlation between maternal PFAS levels and maternal thyroid hormones was evaluated, an interesting sexual dimorphism was found. Indeed, in mothers bearing female fetuses, PFOS and PFOA levels were positively associated with maternal T4 and FTI levels, while there was a negative association for mothers bearing male fetuses. The analysis of the possible correlations between PFAS and cord blood thyroid function parameters consistently showed a direct association between PFAS levels and cord TSH, in particular for PFOS, PFOA, PFDA, PFNA, and FOSA. Finally, the authors evaluated if the effect of PFAS on birth outcomes was mediated by modifications of maternal thyroid function, but the analysis of mediated effects supported a direct role of PFAS on fetal outcomes not mediated by alteration in thyroid function parameters.

One of the main problems in addressing the issue of thyroid disrupting effects consequent to PFAS exposure during pregnancy, is that some evidences suggested a direct detrimental effects of PFAS on the child neurological development, independently from their effects on thyroid function.

Indeed, a positive association between PFAS exposure and several neurological traits such as impulsivity (83), attention-deficit/hyperactivity disorder (ADHD) (84, 85), behavioral problems (86), or poorer executive function (87) was reported by some epidemiological studies. However, these findings were in most cases not confirmed by longitudinal studies, reporting no association between ADHD (88, 89), autism spectrum disorders (88, 90, 91), parent- or teacher-reported behavioral scores (86, 92, 93), or cognitive abilities (94, 95) of exposed children. Some studies showed only weak to moderate associations between child’s behavior and prenatal PFOA, PFNA, and PFDA exposure (96). A recent investigation of nine European cohorts with a total of 4,826 mother-child pairs found overall no associations between prenatal and early postnatal (up to 24 months of age) PFOS and PFOA exposure and ADHD risk (97). Up to now, only a very recent study by Luo et al. addressed the issue of the role of thyroid hormone perturbations as the mechanism through which PFAS would impair neurodevelopment in children (79). In details, Luo et al., evaluated the association between maternal prenatal exposure to six six different PFAS (PFOS, PFOA, PFHxS, PFHpS, PFNA, and PFDA) and behavioral outcomes at 7 and 11 years of age in a cohort of 758 mother-child pairs taken from the Danish National Birth Cohort between 1996 and 2002. Statistical models were adjusted for maternal age, parity, socioeconomic status, pre-pregnancy BMI, gestational week of blood collection, smoking and alcohol intake during the 1st trimester and parent’s behavioral scores. The Authors found a significant association between PFOS, PFOA, PFNA, and PFHxS levels with behavioral difficulties at 7 years of age, but only PFNA levels were associated to behavioral difficulties also at 11 years of age, both in parents- and children-reported measures (79).

The mediation analysis showed that 10–20% of the effect of PFOS, PFOA, PFNA, and PFHxS on the child behavior outcome at 7 years was probably mediated by maternal thyroid hormone alterations, while no mediating effects were observed for any outcome measures at 11 years. The authors concluded that prenatal PFNA exposure was associated with behavioral difficulties in childhood at 7 and 11 years, possibly mediated by maternal thyroid hormones. However, the Authors concluded that the potential slight mediating effects of maternal thyroid hormones in early gestation should be further investigated (79).

## Conclusions

**Figure 1:**
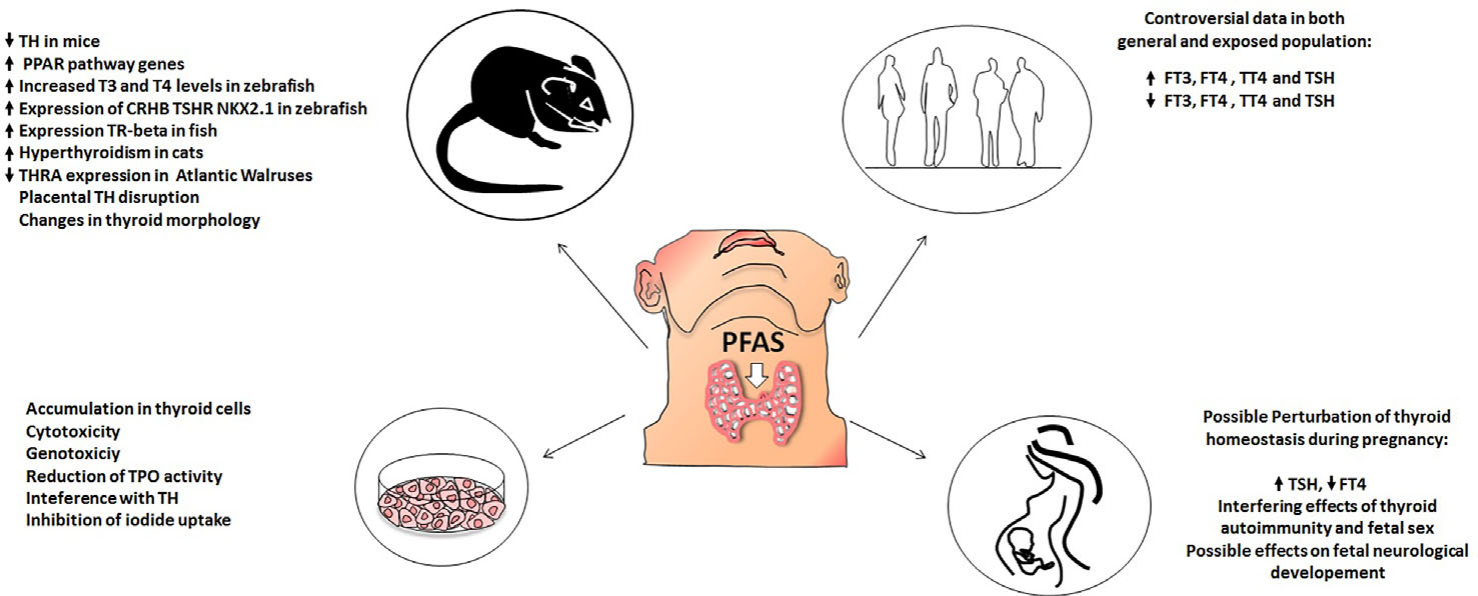
Effect of PFAS on thyroid function in: animal models (upper left), *in vitro* models (lower left), human population (upper right), pregnant women (lower right).

PFAS (both long and short chain and their alternatives) are widespread environmental pollutants. The potential hazards of exposure to these compounds are raising concerns in the scientific community. Among their many detrimental effects, these compounds may act as endocrine disruptors and, at least some of them, are also considered as thyroid disruptors. Although the role of PFAS as thyroid function disrupters is still controversial, *in vitro* studies seem to be in overall agreement regarding the harmful effects of PFAS on thyroid cells. Indeed, PFAS harmful effects on thyroid cells would include accumulation, cytotoxicity, genotoxicity, interference with TH synthesis, TPO function, and iodine uptake, as reported by several by *in vitro* studies. Furthermore, studies performed in animal models confirmed a thyroid disrupting effect of exposure to both new and old generation PFAS. Indeed, PFAS dietary ingestion, induces changes in circulating levels of thyroid hormones, mainly by increasing their metabolic clearance rate. An interesting and worrisome finding is that PFAS accumulate in the placentas of pregnant mice, where they could modify thyroid hormone levels, potentially affecting the development of the fetus; in addition data on zebrafish highlighted an interference of PFAS on embryogenesis. Thus, based on *in vitro* results and on animal models it could be suggested that PFAS exert a thyroid disrupting effect. On the other hand, *in vivo* data in humans are by far more controversial as to the potential thyroid disrupting effect of PFAS with great discrepancy among different studies. At present, the onset of hypothyroidism in PFAS exposed communities as well as in the general population, would represent the most frequent thyroidal effect of the exposure to PFAS, but again with some inconsistencies. Furthermore, PFAS exposure during pregnancy seems to cause slight but statistically significant alterations in thyroid function parameters, both in mothers and in newborns. Still a lot remains to be elucidated regarding the clinical relevance of this phenomenon. Indeed, even if it is probable that PFAS exposure can cause detrimental effects on fetal growth, it is still not clear if this effect is mediated by alterations in thyroid hormone homeostasis. Moreover, long-term studies evaluating the possible neurological consequences of PFAS-related thyroid dysfunction during pregnancy will be needed. **Figure 1** summarizes the available evidence regarding PFAS potential thyroid disrupting effects in different experimental settings (*in vitro* data, animal models, general population, and pregnant women).

## Conclusive Remarks

In conclusion, based on the present review of the literature, we would suggest that available evidence mainly provide by *in vitro* studies and in animal models, would support a thyroid-disrupting effect of the exposure to both old- and new-generation PFAS. However, studies in humans provided contrasting results suggesting the need for more longitudinal, long-term-exposed population studies, specifically designed for evaluating the biological consequences of chronic exposure to PFAS. A further aspect to be addressed will be to evaluate whether exposure to a combination of multiple PFAS could have stronger effects as compared to the exposure to a single PFAS.

## Author Contributions

FC: Conceptualization, Writing, Original draft preparation. LCr: Writing, Original draft preparation. GR: Writing, Table preparation. FM: Reviewing and Editing. MR: Reviewing and Editing, Supervision. MI: Writing, reviewing and editing. LCh: Supervision. All authors contributed to the article and approved the submitted version.

## Conflict of Interest

The authors declare that the research was conducted in the absence of any commercial or financial relationships that could be construed as a potential conflict of interest.

## Abbreviations

PFAS, Per- and polyfluoroalkyl substances; ITRC, Interstate Technology & Regulatory Council; HTP axis, hypothalamic–pituitary–thyroid axis; TRH, thyrotropin-releasing hormone; PFCAs, Perfluoroalkyl carboxylic acids; PFSAs , Perfluoroalkane sulfonic acids; PFOS, Perfluorooctane sulfonic acid; PFOA, Perfluorooctanoic acid; EPA, Environmental Protection Agency; NHANES, National Health and Nutrition Examination Survey; EFSA, European Food Safety Authority; CONTAM, EFSA’s Panel on Contaminants in the Food Chain; ECHA , European Chemicals Agency; PFHxS, Perfluorohexanesulfonic acid; PFBA, Pentafluorobenzoic acid; FTOHs , Fluorotelomer alcohols; GenX; C6O4 , Perfluoro-2-propoxypropanoic acid; Perfluoro{acetic acid, 2-[(5-methoxy-1,3-dioxolan-4-yl)oxy]}; ADONA, 4,8-dioxa-3H-perfluorononanoate; PPARα, Peroxisome-proliferator-activated receptor alpha; SVHC, Substance of Very High Concern; EDCs, Endocrine-Disrupting chemicals; TPO , Thyroperoxidase; TH , Thyroid hormone; THR, Thyroid hormone receptor; PFBS, Perfluorobutane sulfonic acid; PFPA, Perfluoroalkyl phosphonic acids; PFPeA, Perfluoropentanoic acid; TSH, Thyroid-stimulating hormone; cAMP , Cyclic adenosine monophosphate; T4 , Thyroxine; T3, Triiodothyronine; Hpf, Hours post fecondation; PFPiAs, Perfluoroalkyl phosphinic acids; CRH, Corticotropin-releasing hormone; UGT1AB, Uridinediphosphate-glucuronosyltransferase; CRHB, Corticotropin releasing hormone b; TSHR , Thyrotropin receptor; NKX2.1, Thyroid transcription factor-1; TR-β, Thyroid hormone receptor beta; PFNA, Perfluorononanoic acid; PFDA, Perfluorodecanoic acid; PFUnDA, Perfluoroundecanoic acid; MeFOSAA, N-methyl-perfluorooctane sulfonamidoacetic acid; FOSA, Perfluorooctanesulfonamide; EtFOSAA, N-ethyl-perfluorooctane sulfonamidoaceticacid; NR3C1, Nuclear Receptor Subfamily 3 Group C Member 1; THRα, Thyroid hormone receptor alpha; RXRα , Retinoic X receptor alpha; FT4, Free thyroxine; TBG, Thyroxine-binding globulin; PFDoDA, Perfluorododecanoic acid; PFDS , Perfluorodecane sulfonic acid; PFHxA, Perfluorohexanoic acid; BMI, Body mass index; FT3, Free triiodothyronine; *PFHpS*, Perfluoroheptanesulfonic acid; *PFOSA*, Perfluorooctanesulfonamide; Ab , Antibody; PFTrDA , Perfluorotridecanoic acid; Tg, Thyroglobulin; HOME; ADHD, Health Outcomes and Measures of the Environment; Attention-deficit/hyperactivity disorder; BKMR, Bayesian kernel machine regression; ORs, Odd ratios; FTI, Free T4 index; NIS , Sodium/iodide symporter.

## Glossary

**Table:**

PFAS	Per- and polyfluoroalkyl substances
ITRC	Interstate Technology & Regulatory Council
HTP axis	hypothalamic–pituitary–thyroid axis
TRH	thyrotropin-releasing hormone
PFCAs	Perfluoroalkyl carboxylic acids
PFSAs	Perfluoroalkane sulfonic acids
PFOS	Perfluorooctane sulfonic acid
PFOA	Perfluorooctanoic acid
EPA	Environmental Protection Agency
NHANES	National Health and Nutrition Examination Survey
EFSA	European Food Safety Authority
CONTAM	EFSA’s Panel on Contaminants in the Food Chain
ECHA	European Chemicals Agency
PFHxS	Perfluorohexanesulfonic acid
PFBA	Pentafluorobenzoic acid
FTOHs	Fluorotelomer alcohols
GenX	C6O4
Perfluoro-2-propoxypropanoic acid	Perfluoro{acetic acid, 2-[(5-methoxy-1,3-dioxolan-4-yl)oxy]}
ADONA	4,8-dioxa-3H-perfluorononanoate
PPARα	Peroxisome-proliferator-activated receptor alpha
SVHC	Substance of Very High Concern
EDCs	Endocrine-Disrupting chemicals
TPO	Thyroperoxidase
TH	Thyroid hormone
THR	Thyroid hormone receptor
PFBS	Perfluorobutane sulfonic acid
PFPA	Perfluoroalkyl phosphonic acids
PFPeA	Perfluoropentanoic acid
TSH	Thyroid-stimulating hormone
Camp	Cyclic adenosine monophosphate
T4	Thyroxine
T3	Triiodothyronine
Hpf	Hours post fecondation
PFPiAs	Perfluoroalkyl phosphinic acids
CRH	Corticotropin-releasing hormone
UGT1AB	Uridinediphosphate-glucuronosyltransferase
CRHB	Corticotropin releasing hormone b
TSHR	Thyrotropin receptor
NKX2.1	Thyroid transcription factor-1
TR-β	Thyroid hormone receptor beta
PFNA	Perfluorononanoic acid
PFDA	Perfluorodecanoic acid
PFUnDA	Perfluoroundecanoic acid
MeFOSAA	N-methyl-perfluorooctane sulfonamidoacetic acid
FOSA	Perfluorooctanesulfonamide
EtFOSAA	N-ethyl-perfluorooctane sulfonamidoaceticacid
NR3C1	Nuclear Receptor Subfamily 3 Group C Member 1
THRα	Thyroid hormone receptor alpha
RXRα	Retinoic X receptor alpha
FT4	Free thyroxine
TBG	Thyroxine-binding globulin
PFDoDA	Perfluorododecanoic acid
PFDS	Perfluorodecane sulfonic acid
PFHxA	Perfluorohexanoic acid
BMI	Body mass index
FT3	Free triiodothyronine
*PFHpS*	Perfluoroheptanesulfonic acid
*PFOSA*	Perfluorooctanesulfonamide
Ab	Antibody
PFTrDA	Perfluorotridecanoic acid
Tg	Thyroglobulin
HOME, ADHD	Health Outcomes and Measures of the Environment; Attention-deficit/hyperactivity disorder
BKMR	Bayesian kernel machine regression
Ors	Odd ratios
FTI	Free T4 index
NIS	Sodium/iodide symporter.
